# Vaccine antigens modulate the innate response of monocytes to Al(OH)_3_

**DOI:** 10.1371/journal.pone.0197885

**Published:** 2018-05-29

**Authors:** Sietske Kooijman, Jolanda Brummelman, Cécile A. C. M. van Els, Fabio Marino, Albert J. R. Heck, Elly van Riet, Bernard Metz, Gideon F. A. Kersten, Jeroen L. A. Pennings, Hugo D. Meiring

**Affiliations:** 1 Intravacc, Bilthoven, The Netherlands; 2 Biomolecular Mass Spectrometry and Proteomics, Bijvoet Center for Biomolecular Research and Utrecht Institute for Pharmaceutical Sciences, Science Faculty, Utrecht University, Utrecht, The Netherlands; 3 Centre for Infectious Disease Control, National Institute for Public Health and the Environment, Bilthoven, The Netherlands; 4 Netherlands Proteomics Centre, Utrecht, The Netherlands; 5 Leiden Academic Centre for Drug Research, Leiden University, Leiden, The Netherlands; 6 Centre for Health Protection, National Institute for Public Health and the Environment, Bilthoven, The Netherlands; National Institutes of Health, UNITED STATES

## Abstract

Aluminum-based adjuvants have widely been used in human vaccines since 1926. In the absence of antigens, aluminum-based adjuvants can initiate the inflammatory preparedness of innate cells, yet the impact of antigens on this response has not been investigated so far. In this study, we address the modulating effect of vaccine antigens on the monocyte-derived innate response by comparing processes initiated by Al(OH)_3_ and by Infanrix, an Al(OH)_3_-adjuvanted trivalent combination vaccine (DTaP), containing diphtheria toxoid (D), tetanus toxoid (T) and acellular pertussis (aP) vaccine antigens. A systems-wide analysis of stimulated monocytes was performed in which full proteome analysis was combined with targeted transcriptome analysis and cytokine analysis. This comprehensive study revealed four major differences in the monocyte response, between plain Al(OH)_3_ and DTaP stimulation conditions: (I) DTaP increased the anti-inflammatory cytokine IL-10, whereas Al(OH)_3_ did not; (II) Al(OH)_3_ increased the gene expression of *IFNγ*, *IL-2* and *IL-17a* in contrast to the limited induction or even downregulation by DTaP; (III) increased expression of type I interferons-induced proteins was not observed upon DTaP stimulation, but was observed upon Al(OH)_3_ stimulation; (IV) opposing regulation of protein localization pathways was observed for Al(OH)_3_ and DTaP stimulation, related to the induction of exocytosis by Al(OH)_3_ alone. This study highlights that vaccine antigens can antagonize Al(OH)_3_-induced programming of the innate immune responses at the monocyte level.

## Introduction

In 1926, the adjuvant features of colloidal aluminum salts were discovered by observing that diphtheria toxoid adsorbed to aluminum induced a significantly higher antibody titer against the toxoid than the antigen alone [1]. Since the discovery of their adjuvant activity, aluminum salts have been widely used as vaccine adjuvants in human vaccines. For a long time the mechanism of action of aluminum adjuvants was largely unknown. Based on the observation that aluminum salt-adsorbed toxoids were cleared more slowly from the injection site than non-adsorbed toxoids [2], it was hypothesized that antigen-aluminum salts act as a depot at the site of injection, causing a slow antigen release. However, in guinea pig experiments, the immune response was not compromised when the salt deposit was removed from the injection site [2, 3], indicating that this is at least not the only mechanism by which Al(OH)_3_ affects the immune response towards antigens. Other modes of action suggested for the adjuvant effect of Al(OH)_3_ include early innate mechanisms such as differentiation of monocytes to antigen-presenting dendritic cells [4], triggering and recognition of Danger Associated Molecular Patterns (DAMPs) [5–7] promoting T-helper (Th) 2 differentiation [4, 8], recruitment of immune cells to the site of injection [9, 10], inflammasome activation [7, 11, 12], complement activation [13], increasing antigen presentation via HLA class I and II [14], enhanced phagocytosis [15].

Most of the previous studies analyzed the effect of Al(OH)_3_ with either Al(OH)_3_ [4, 14, 16] alone or in combination with a model antigen like ovalbumin or alpha casein [7, 8, 11, 15]. Since antigens may have a distinct effect on innate immune cells, the question arises to what extent particular antigens skew the innate effects of the adjuvant.

DTaP vaccine was implemented for active immunization against diphtheria, tetanus and pertussis in infants and children. Besides diphtheria toxoid and tetanus toxoid, DTaP comprises *Bordetella pertussis*-derived filamentous hemagglutinin, pertussis toxoid and pertactin P.69 antigens. DTaP vaccination typically induces a Th2-biased and regulatory T cell response [15, 17, 18] not optimally conferring long-term protective immunity to pertussis. For vaccine development it is important to understand whether antigens affect the innate phase of the immune response or the instructions of the adaptive response of the adjuvant, or both.

In this study, we compared the innate immune responses induced by Al(OH)_3_ alone *versus* that of a licensed combination DTaP vaccine containing Al(OH)_3_, using cytokine analysis, transcriptomics and proteomics, to determine unique, shared and potential synergistic or antagonistic effects of adjuvant and antigen components. Primary human monocytes were used as the main innate cell platform since these are prominent mononuclear phagocytes in the blood and play, when activated, an important role in bridging the innate and adaptive immune response in tissue [19, 20]. Besides this, their known differentiation into monocyte-derived dendritic cells (MoDCs) monocyte can also enforce their antigen presenting role to T cells in response to various stimuli [21]. This systems-based approach results in a comprehensive insight in the molecular pathways involved in innate immune activation of monocytes upon stimulation with Al(OH)_3_-based vaccines.

## Materials and methods

### Ethics statement

This study was conducted according to the principles expressed in the Declaration of Helsinki. Written informed consent was obtained from all blood donors before collection and use of their samples. All blood samples were processed anonymously. All blood donations, provided by the Dutch National Institute for Public Health and the Environment (RIVM, Bilthoven; The Netherlands), were specifically given for primary cell isolation, a research goal explicitly approved by the accredited Medical Research Ethics Committee (MREC), METC, Noord-Holland in The Netherlands.

### Materials used for cell stimulation

Aluminum hydroxide (Al(OH)_3_) Alhydrogel 2%, Brenntag (Frederikssund; Denmark) was used as the adjuvant. The registered DTaP vaccine Infanrix was obtained from GlaxoSmithKline (Brentford; Middlesex; UK). The vaccine contains combined antigens, *i*.*e*. a minimum of 30 international units (I.U.) diphtheria toxoid, a minimum of 40 I.U. tetanus toxoid, 25 μg filamentous hemagglutinin (FHA), 25 μg pertussis toxoid, and 8 μg pertactin P.69 (PRN), all absorbed to 0.5 mg of Al(OH)_3_ per dose. LPS from *E*.*coli* K12 (Invivogen; San Diego; California; USA) was used as positive control. Non-adsorbed *Bordetella pertussis* antigens PRN and Pertussis toxin (PTx) were obtained from Biotrend (Cologne; Germany) and FHA was purchased from Sigma Aldrich (Darmstadt; Germany).

### Monocyte isolation and stimulation

Blood from 5 healthy adult donors was used for Peripheral blood mononuclear cell (PBMC) isolation and subsequent monocyte isolation. PBMCs were obtained by density gradient centrifugation on Lymphoprep (Nycomed; Zurich; Switzerland) at 1,000*x*g for 30 minutes. Subsequently, monocytes were isolated from the obtained PBMC fraction using anti-CD14 MACS beads in combination with MACS (Miltenyi Biotech; Bergisch Gladbach; Germany). A purity check of the monocytes was performed by flow cytometric analysis of CD14 cell surface expression and only if the purity of the monocyte population was ≥95% the cells were used for proteome and transcriptome analysis.

The isolated monocytes, 600,000 cells/well were cultured in a 24-wells plate (Corning; Corning; New York; USA) in 1.5 ml of RPMI (Gibco/Thermo Fisher; Waltham; Massachusetts; USA) containing 10% Fetal Calf Serum (FSC) (Hyclone), 100 units/ml of penicillin (Gibco) 100 units/ml streptomycin (Gibco) and 2.92 mg/ml L-glutamin (Gibco) (culture medium). Monocytes were either left unstimulated or were stimulated with a final concentration of 0.1 μg/ml LPS, 10 μg/ml Al(OH)_3_ or DTaP containing a final concentration of 10 μg/ml Al(OH)_3_ per stimulation condition per donor. After 24 and 48 hours of stimulation, culture supernatants were collected for cytokine assays and monocytes were harvested for targeted transcriptome and whole proteome analysis. For both proteomics and gene expression analysis, the material of three individual donors was available. LPS was used as a positive control for the cell culture and only when LPS performed as expected, by inducing CD80, as described previously [16] (S1 Fig), the samples were used.

### Culture and stimulation of THP-1 cells

A human monocytic cell line THP-1 (ATCC; Teddington; Middlesex; U.K.), was used to verify pathways or leads identified in primary monocytes, *i*.*e*. Inflammasome activation and IL-10 secretion.

THP-1 cells were cultured according to the supplier’s protocol. To investigate the IL-1β secretion induced by Al(OH)_3_ or DTaP, the cells were primed with 300 ng/ml phorbol 12-myristate (PMA) (Sigma-Aldrich; Darmstadt, Germany) for 24 hours. After 24 hours of priming, the cells were placed in culture medium without PMA for 24 hours [16]. The medium was refreshed again and cells were either left unstimulated or stimulated with 50 μg/ml DTaP or the same concentration of Al(OH)_3_ (based on a dose response curve in THP-1 cells), in the presence or absence of 25 μg/ml of an inflammasome blocker (Glybenclamide) (Invivogen; San Diego; California; USA) for 48 hours.

For determination of the IL-10-inducing component, THP-1 cells were stimulated with 50 or 100 μg/ml Al(OH)_3_ alone or the same concentrations of Al(OH)_3_ in DTaP or with PTx (0.165 μg/ml to 5.28 μg/ml), FHA (0.165 μg/ml to 5.28 μg/ml), PRN (0.05 μg/ml to 1.6 μg/ml) alone all in a twofold dilution series. The concentration range used was based on the concentrations of the individual antigens in the complete vaccine. Synergy in a complete vaccine cannot be excluded; therefore the concentration range started just below the lowest vaccine dose used in THP-1 cell stimulations. The DTaP stimulations contained 0.33 or 0.66 μg/ml of PTx or 0.33 or 0.66 μg/ml of FHA or 0.2 or 0.4 μg/ml of PRN, respectively. Cells were stimulated for 48 hours. Supernatants were analyzed for the presence of IL-10 by ELISA.

### mRNA expression analysis

mRNA Isolation from monocytes (from three different donors) was performed using the RNeasy mini kit (Qiagen; Venlo; The Netherlands), according to the manufacturer’s animal cell spin protocol. RNA purity and concentration was determined using spectrophotometric analysis of the 260-nm and 280-nm absorbance, on the NanoDrop 2000 (Thermo Fisher; Waltham MA; USA). cDNA synthesis of 12 ng of RNA was performed using the RT cDNA synthesis kit and the RT preAMP pathway primer mix innate and adaptive immunity (both from Qiagen; Venlo; The Netherlands); cDNA was frozen at -20°C. qPCR measurements were performed using the Roche Light Cycler 96 (Roche; Basel; Switzerland) and the innate and adaptive immune response RT^2^ profiler arrays (Qiagen; Venlo; The Netherlands), comprising 89 functional genes and 7 controls. A melt curve determination was included in the measurement for quality control [16].

Gene expression of each donor was normalized to the three most stable housekeeping genes ACTB, HPRT1 and RPLP0. After normalization, the fold-change was determined, meaning normalized gene expression (2^-ΔCt^) in the test sample divided by the normalized gene expression (2^-ΔCt^) in the control sample. Fold change values greater than one indicates an up-regulation of 2^1^ or more. Fold-change values less than one indicate down-regulation. Genes were considered regulated when they differed a factor 2 or more from the control in two out of three donors, based on the SD between technical replicates of 0.13*x*Ct values, corresponding to a coefficient of variation (CV) of 9.4% (S1 Table). This means that a two-fold change in gene expression more than three times exceeds the CV and is a meaningful difference.

### Protein isolation, digestion and labeling

To isolate the proteins from the monocytes (from 3 three different donors) the cells were incubated with 500 μl of 4 M guanidine·HCl in phosphate buffer, pH 7.5, at 4°C for two hours. During incubation, the cells were subjected to a freeze-thaw step. After the cell lysis, 50 μl of the lysate of each sample was used to determine the protein concentration using the BCA protein assay (Pierce Biotechnology; Waltham; Massachusetts; USA), according to the manufacturer’s protocol. The remaining lysed cells were stored at -80°C.

To reduce the guanidine·HCl concentration, protein samples were diluted four times with 100 mM phosphate buffer pH 7.5. Subsequently, the proteins were digested with Lys-C (Roche) in an enzyme-to-substrate ratio of 1:10 (w/w) at 37°C. After 4 hours, fresh Lys-C was added in a 1:10 (w/w) enzyme-to-substrate ratio for an overnight incubation.

Normalization on protein content was performed on aliquots of the digested protein samples from the 6 conditions (medium, Al(OH)_3_ and DTaP all at 24 and 48 hours) per individual donor. The samples were labeled per condition on solid phase extraction (SPE) columns (Waters; Milford; MA; USA) using tandem mass tag labeling-6plex (TMT(6), Thermo Fisher). The SPE columns were equilibrated as described by the manufacturer. Columns were washed with 100 mM phosphate buffer pH 7.5.

The digested samples were loaded onto individual SPE columns per condition, using a vacuum manifold (Waters), after which samples were washed with 100 mM phosphate buffer pH 7.5. The labeling reagent (TMT(6)) was reconstituted in acetonitrile (AcN) according to the supplier’s protocol (0.8 mg per individual label in 41 μl AcN), after which the AcN concentration was reduced to a maximum of 2.5% (v/v) with 100 mM phosphate buffer pH 7.5. The individual TMT(6) labels were loaded onto the 6 individual SPE columns leaving 0.5 ml reagent on top of the column for a 30 minute incubation. After 30 minutes, fresh label was added for another 30 minutes of incubation subsequently, the columns were washed with water containing 0.5% formic acid (FA). The individual stimulation conditions per donor were eluted from the column with 90% Acetonitrile (AcN) containing 0.5% formic acid (FA), were pooled and then dried by centrifugation under reduced pressure and reconstituted in Trifluoroacetic acid (0.1%TFA).

### Peptide fractionation by SCX

To purify and fractionate the labeled monocyte-derived digested protein samples, Strong Cation eXchange (SCX) was used as described previously [22]. The system comprised an in-house made Hypercarb trapping column (200 μm I.D. x 5 mm length, 7 μm particle size) and an in-house made SCX column (200 μm I.D. x 11 cm length PolySULFOETHYL Aspartamide, 5 μm, PolyLC). Elution was 12 min at 100% solvent A (water + 0.5% HOAc) followed by a 16.5 minute linear gradient to 100% solvent B (250 mM KCl + 35% AcN+ 0.5% HOAc in water) and a second linear gradient of 16.5 min to 100% solvent C (500 mM KCl + 35% AcN+ 0.5% HOAc in water). Twenty-six SCX fractions were obtained and of each 4 μL was subjected to nanoscale LC-MS analysis.

### LC-MS/MS analysis

Peptide separation of the individual SCX fractions was performed on a Proxeon Easy-nLC 1000 system (Thermo Scientific; San Jose; CA; USA). Peptides were trapped on a double-fritted trapping column Reprosil (Dr. Maisch; Ammerbuch; Germany) C18; df = 3 μm, 2 cm length × 100 μm I.D., made in-house and separated on an in-house-packed analytical column Poroshell (Agilent; Waldbron; Germany) 120 EC-C18; df = 2.7 μm, 50 cm length × 50 μm I.D.), at a column temperature of 40°C. Solvent A was MilliQ water containing 0.1% FA and solvent B was 0.1% FA in AcN (Biosolve). The peptides were separated in 133 minutes (10 minutes at 2% B, from 2% to 30% B in 118 minutes and 5 minutes at 70% B) in a non-linear gradient optimized as described by Morus *et al*. [23]. After 133 minutes, the system was kept at 5% B for 15 minutes to equilibrate the column for the next injection. The column effluent was electro-sprayed directly into the MS using a gold-coated fused silica tapered tip of 5 μm, at a spray voltage of 1.8 kV.

Mass spectrometric data were acquired on a Tribrid-Orbitrap Fusion (Thermo Fisher Scientific; San Jose; CA; USA). The full scan (MS^1^) spectra were acquired with a scan mass range of *m/z* 350–1500 at 120,000 resolution (FWHM) with an Orbitrap readout. For the MS^1^ the maximum injection time was 50 ms and the automatic gain control (AGC) was set to 200,000. Top speed mode was chosen with a duration of 3 s where precursor ions with an intensity >5,000 were selected for fragmentation (MS^2^). For MS^2^ charge states between 1 and 7 were selected. MS^2^ was performed using Collision-Induced Dissociation (CID) in the linear ion trap (LTQ) with a normalized collision energy of 35%. In MS^2^ the AGC was set to 10,000 and the maximum injection time was 35 ms. Synchronous-Precursor-Selection (SPS) was used to include up to 10 MS^2^ fragment ions in MS^3^. These fragment ions were further fragmented by Higher energy Collision Dissociation (HCD) with a normalized collision energy of 50%. The TMT reporter ions were analyzed in the Orbitrap with a maximum injection time of 120 ms, and an AGC of 100,000.

Proteomics data were analyzed with Proteome Discoverer 1.4, (Thermo Fisher Scientific; San Jose; CA; USA). Default settings were used, unless stated otherwise. Precursor mass tolerance was set to 5 ppm. MS^2^ scans were searched, with the Sequest HT search engine and a full enzyme specificity for Lys-C, against the human Uniprot database from November 2014, containing 23,048 entries. *b*-Type ions and *y*-type ions were enabled for CID and HCD data using a fragment mass tolerance of 0.5 Da. The quantification node was used to obtain relative expression values, where TMT(6) was defined as the quantification method, with an integration tolerance of 0.2 Da. Percolator was used to filter the peptide to spectrum mass with a false discovery rate (FDR) of <5%. The data was searched with Aspargine deamidation and Methionine oxidation as dynamic modifications and TMT(6) was set as a static modification on the N-termini and Lysine residues.

The results of the separate SCX fractions were integrated in the data analysis for each individual donor. When multiple entries occurred, based on Uniprot and NCBI data for the same protein, the ratios as provided by Proteome Discoverer were Log2-transformed and these entries were averaged for further analysis. Next, data were normalized by performing a median correction. Data of three individual donors were compared: proteins that were upregulated or downregulated by 1.5 fold or more compared to control in at least two out of three biological replicates were considered regulated. The fold change of 1.5 was based upon being 3 times the median coefficient of variation (CV) of the technical variation, which corresponds to a *p-*value of < 0.01. The regulated proteins were imported in STRING (string.embl.de) [24] and Protein Center to identify enriched pathways (FDR<0.1), within functional annotations provided by Gene Ontology (GO) biological processes and Kyoto Encyclopedia of Genes and Genomes (KEGG) using the following String settings: medium confidence and the interactions sources: experiments, co- expression, co-occurrence and database.

Venn diagrams were created using http://bioinfogp.cnb.csic.es/tools/venny/ with the regulated proteins for each condition and time point incorporated.

Protein network was created using Cytoscape based on the enriched pathways in each up or downregulated protein set. String network analysis of the gene expression data was performed.

### Cytokine ELISA

In the supernatants of THP-1 cultures, IL-1β was determined by using the Human IL-1 beta/IL-1F2 DuoSet ELISA (R&D systems; McKinley; Minneapolis; USA) and IL-10 by human IL-10 ELISA Ready set go (Affymetrix; eBiosciences; San Diego; CA; USA), both according to the manufacturer’s protocol. Samples were measured on a Synergy MX (Biotek; Winooski; Vermont; USA). ELISA data were analyzed with Graphpad Prism^®^. Significance of difference between stimulation conditions was determined using multiple T-tests one per row with the FDR approach with the two-stage linear setup procedure of Benjamini, Q = 5%.

## Results

### DTaP, like Al(OH)_3_, induces differentiation of monocytes

**Fig 1:**
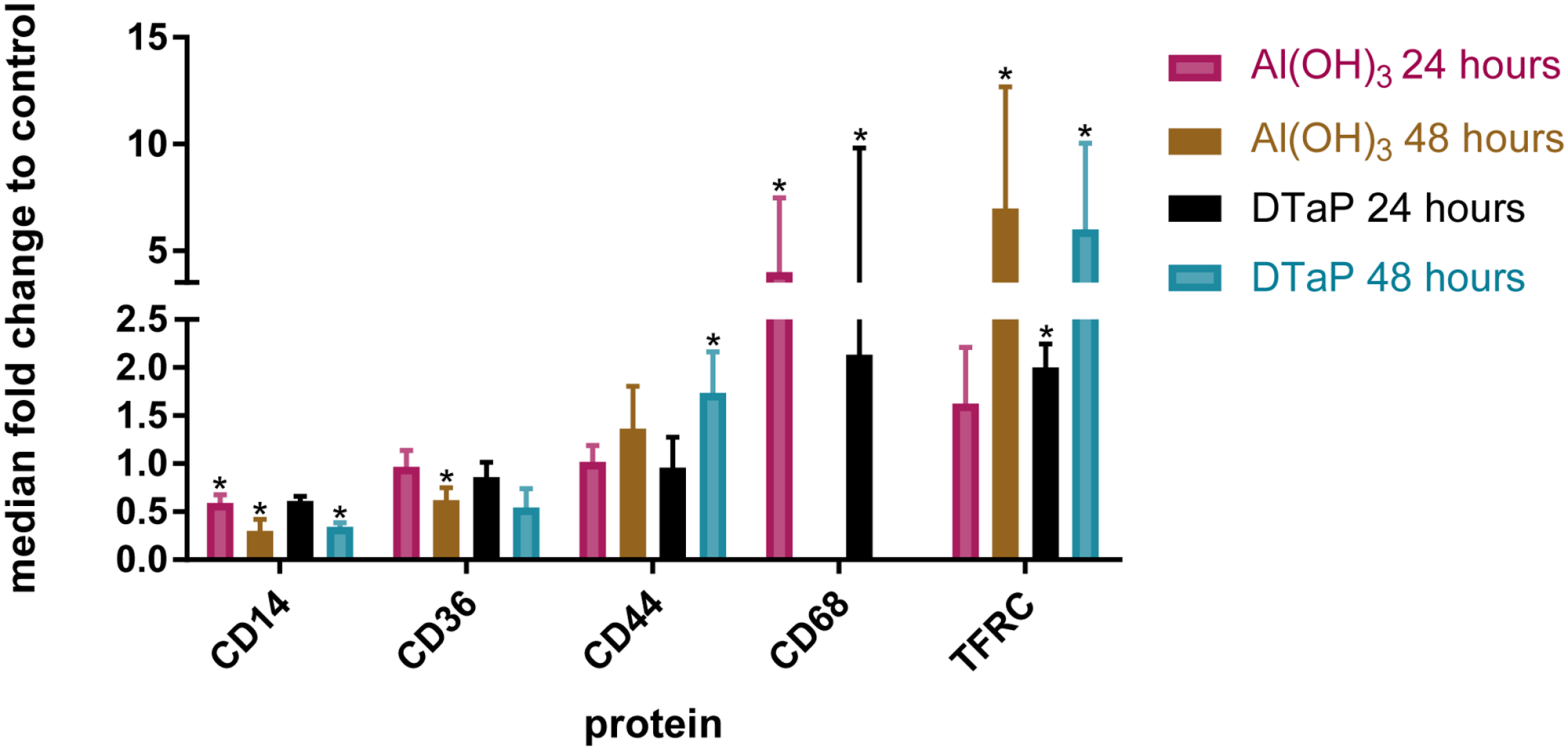
Expression profiles of cell surface proteins of stimulated monocytes as determined by mass spectrometry. Fold changes (average and range) of the expression at the protein level of indicated cell surface markers by monocytes under the indicated stimulation conditions. Both contain 10 μg of Al(OH)_3_. The absence of a bar means that this marker was quantified not more than once for this stimulation condition. Significance of difference compared to control were determined with a student *t*-test with Bonferroni set up *p-*values <0.05 are depicted with an *.

**Fig 2:**
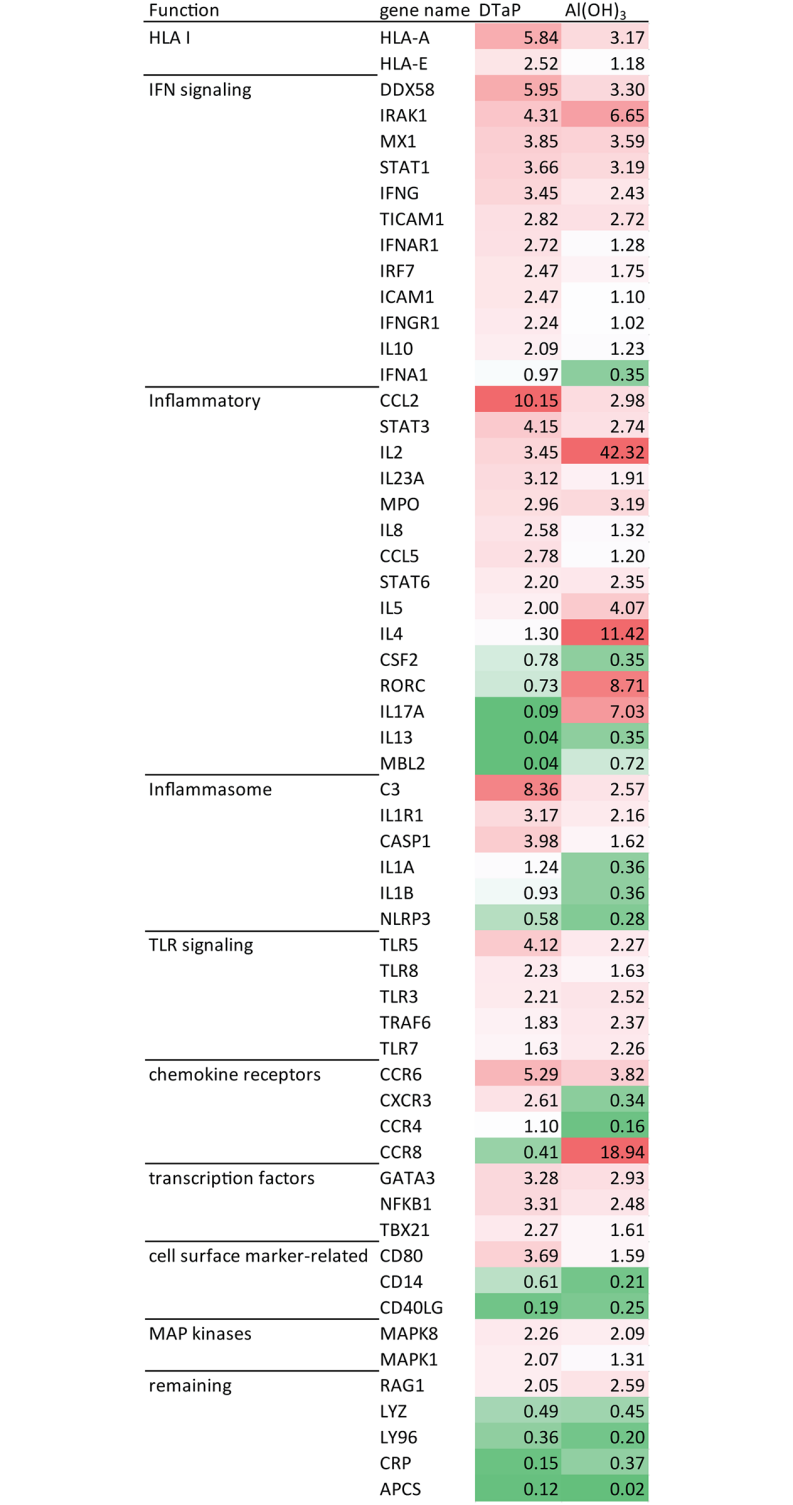
Gene expression after 24 h of stimulation of monocytes. Regulated genes in monocytes (at least a factor 2 up or down) are depicted after 24 hours of Al(OH)_3_ or DTaP stimulation. The median fold change from the 3 donors is depicted. The genes are clustered in a heatmap based on their molecular function.

To first assess whether primary monocytes show characteristics of activation and differentiation when stimulated with the Al(OH)_3_-adjunvanted vaccine DTaP or with Al(OH)_3_ alone, the expression of known cell surface markers was surveyed selectively with targeted transcriptome analysis and unbiased by mass spectrometry-based proteomics. Al(OH)_3_ increased the expression of activation markers, at the protein level [16]. The activation marker TFRC (*i*.*e*. CD71, after 48 hours) was increased at least two fold in both Al(OH)_3_ and DTaP-stimulated monocytes (Fig 1, Table 1). Another protein indicating an active function of the monocytes, CD44 (involved in the adhesion of leukocytes), was significantly upregulated in DTaP-stimulated cells, after 48 hours. In addition, the gene expression of the costimulatory marker *CD80* was induced by DTaP also when compared to plain Al(OH)_3_, after 24 hours of stimulation [16] (Fig 2).

**Table 1:** Proteins involved in the processes described and their median fold changes after 24 and 48 hours of stimulation of three donors.

Protein	Fold changes			
	DTAP stimulation		Al(OH)_3_ stimulation	
	24 hours	48 hours	24 hours	48 hours
HLA-A	*0*.*85*	1.95	*1*.*04*	2.47
HLA-E	1.84	N.D.	1.75	N.D.
HSP90AA1	*1*.*16*	*1*.*29*	1.60	*1*.*31*
Minor histocompatibility antigen H13	*1*.*06*	1.68	N.D.	2.01
Full-length cDNA clone CS0DI002YH20 of Placenta of Homo sapiens (human) Legumain	*1*.*43*	1.75	2.09	1.83
Cathepsin B	*1*.*27*	1.95	*1*.*39*	*0*.*99*
Cathepsin D	2.11	2.50	2.73	2.16
Cathepsin L1	2.95	2.48	4.66	2.29
Cathepsin S	*1*.*11*	1.73	*1*.*14*	1.78
CD9 antigen	N.D.	1.4	1.28	1.61
Monocyte differentiation antigen CD14, urinary form CD14*	*0*.*69*	*0*.*44*	*0*.*68*	*0*.*44*
Macrosialin	2.12	N.D.	3.13	N.D.
CD71 (TFRC)	2.00	4.96	1.70	5.75
MX1	*1*.*07*	1.99	1.50	1.64
MX2	*1*.*00*	*1*.*32*	*1*.*23*	1.90
IFI30	1.61	*1*.*39*	2.33	*1*.*4*
IFIT2	*1*.*03*	*1*.*44*	N.D.	*1*.*44*
IFIT3	*1*.*05*	2.13	1.64	2.62
IL3RA	1.73	1.55	2.22	1.61
IFNγR1	*1*.*03*	*1*.*29*	1.90	3.08
IRF5	*1*.*06*	1.73	*1*.*15*	*1*.*21*
C4	*0*.*78*	2.31	1.82	1.65
C5aR	1.57	2.19	*1*.*48*	2.10
C8a	1.75	*0*.*95*	1.87	*1*.*48*

* CD14 was only identified in one donor twice.

N.D. there were no quantitative data at this time point.

Median fold change of three biological replicates of monocytes: a fold change ≥1.5 compared to medium-stimulated monocytes was considered significant. Non-significant genes are depicted in *italic*.

The expression of the monocyte differentiation antigen CD14 [25, 26], was downregulated after 48 hours of Al(OH)_3_ stimulation [16]. This was also the case in response to the Al(OH)_3_-containing vaccine DTaP after 48 hours of stimulation (Fig 1, Table 1). The loss of CD14 indicates that both DTaP-stimulated monocytes and Al(OH)_3_-stimulated monocytes differentiate away from a monocytic cell type.

### Quantitative proteomics reveals distinct protein expression and pathway enrichments in monocytes induced by DTaP compared to Al(OH)_3_

**Fig 3:**
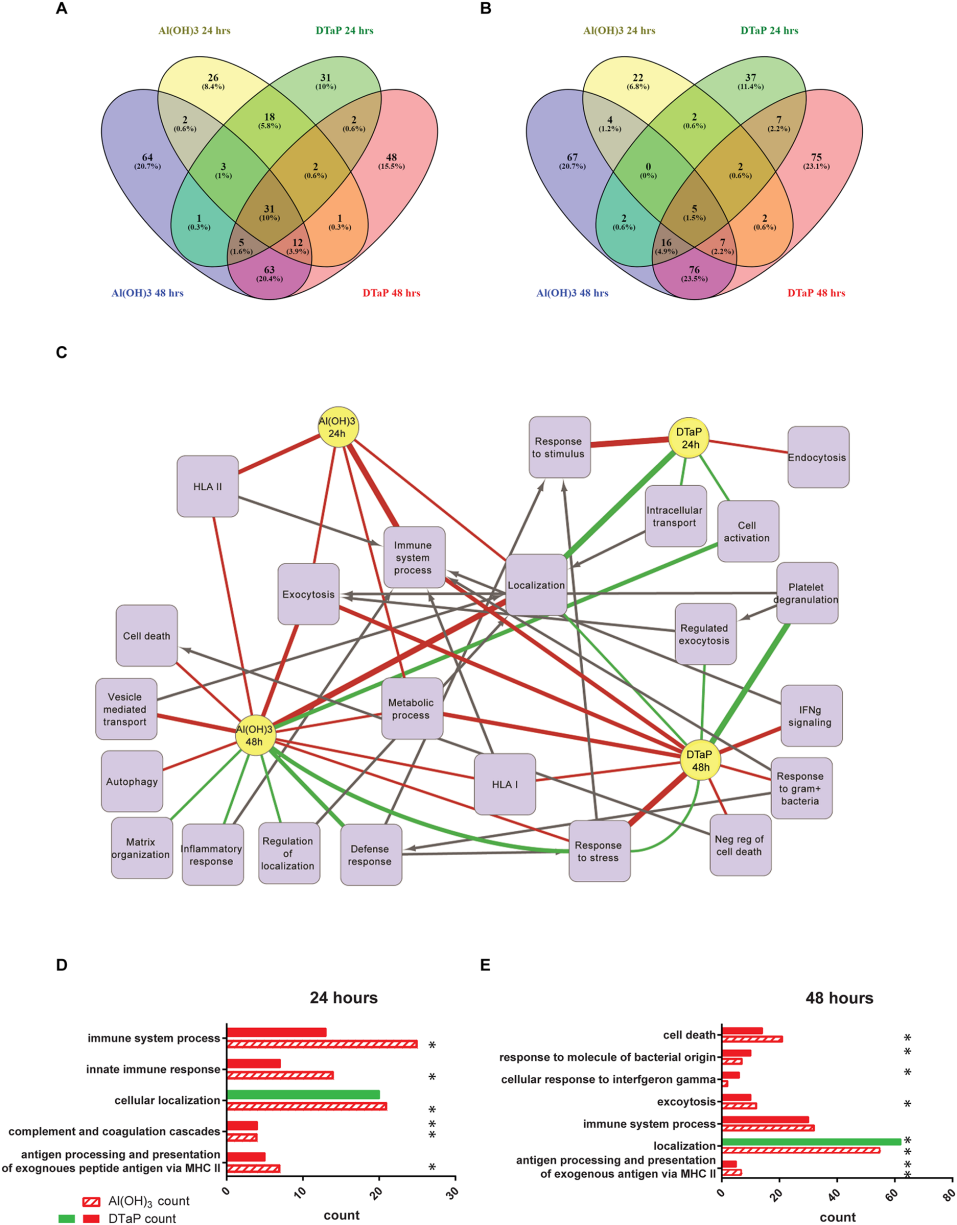
Venn diagram and protein network analysis of regulated proteins. Venn diagrams indicate numbers of sharing and upregulated (A) and downregulated (B) proteins in DTaP *versus* Al(OH)_3_ adjuvant-stimulated monocytes after 24 hours and 48 hours. Purple represents Al(OH)_3_ 24 hours, yellow represents DTaP 24 hours, green represents Al(OH)_3_ 48 hours and red represents DTaP 48 hours. All proteins depicted in these Venn diagrams were regulated by a factor of at least 1.5 in at least two out of three donors. Up and downregulated proteins, identified per stimulation condition and per time point by mass spectrometric analysis, were assessed based on Gene Ontology biological process enrichment (GO terms). An overview of the main enriched pathways is depicted in a protein network (C). The yellow circles represent the stimulation conditions, the purple squares represent the processes. Green lines from a condition towards a process represent a downregulated pathways. Red lines from a condition towards a process represent an enrichment of these processes in the upregulated protein sets. The width of the line represents the significance of the enrichment factor: the thicker the line the more significantly enriched the process; the thinnest lines represent a *p*-value <0.05, the medium lines represent a *p*-value <0.01 and the thickest lines represent a *p*-value <0.001. The black arrows connect daughter terms with the mother term. All terms are at least enriched with a False Discovery Rate *p* value of <0.05. Bar graphs of the number of proteins found to be regulated upon the stimulation conditions after 24 hours (D) and 48 hours (E), in which red represents that process was enriched in the upregulated proteins set and green represents that the process is enriched in the downregulated protein set. The * depicts the significantly regulated pathways.

Quantitative proteomics of Al(OH)_3_ and DTaP-stimulated monocytes resulted in the identification of 4,000 unique proteins of which 3,000 proteins were relatively quantified. 650 Proteins were regulated as a result of one of the stimulation conditions compared to unstimulated control (Fig 3A and 3B, S2 Table). Proteins were clustered in GO terms and KEGG pathways and the differences in these terms and pathways between the stimulations and control were identified with a pathway overrepresentation analysis (S3 Table). It was previously observed that after 24 hours of stimulation with Al(OH)_3_, several immunological relevant GO terms were overrepresented [16]. In addition, localization processes were also enriched [16]. Also processes requiring localization such as, *vesicle mediated transport* and *exocytosis* were enriched (Fig 3C, S3 Table). After 48 hours of Al(OH)_3_ stimulation, the immune system-related pathways were still enriched, as were the localization processes and the processes requiring localization, *e*.*g*. *secretion by cell* and *exocytosis*. The inflammatory response was downregulated after 48 hours of Al(OH)_3_ stimulation [16] (Table 2, Fig 3C, S3 Table).

**Table 2:** Overview of enriched processes identified by quantitative proteomics extracted from S3 Table.

DTaP up 24 hrs	DTaP up 48 hrs	DTaP down 24 hrs	DTaP down 48 hrs	Al(OH)_3_ up 24 hrs	Al(OH)_3_ up 48 hrs	Al(OH)_3_ down 24 hrs	Al(OH)_3_ down 48 hrs
Endocytosis	Endocytosis	Endocytosis	Coagulation	Lysosome	Lysosme	None	Inflammatory response
Response to stimulus	Defense response	Cell activation	Localization	Immune system process	Immune system process		Defense response
Complement and coagulation cascades	Antigen processing and presentation	Regulation of T cell activation	Response to stress	Antigen processing and presentation	Antigen processing and presentation		Response to stress
	Exocytosis	Platelet activation	Exocytosis	Exocytosis	Exocytosis		
	Immune response	Transport		Transport	Transport		
	Cell proliferation	Localization		Localization	Localization		
	Cytokine-mediated signaling pathway			Metabolic processes	Metabolic pathways		
	Autophagy			Catabolic processes	Regulation of cell death		
	Translation			Complement and coagulation cascades	Vesicle-mediated transport		
	Interferon-gamma-mediated signaling pathway			Valine, leucine and isoleucine biosynthesis			

In contrast, upon 24 hours of DTaP stimulation (adjuvanted with Al(OH)_3_) no immune response-related GO terms were overrepresented (Table 2, S3 Table). Amongst the 4 KEGG pathways identified, one immune system-related KEGG pathway was found: *activation of complement* and *coagulation pathway*s (S3 Table). *Endocytosis* was overrepresented in both the up and downregulated protein set (Table 2, Fig 3C and 3D, S3 Table). The downregulated protein set overrepresented *localization pathways* (Table 2, Fig 3C, S3 Table). After 48 hours of DTaP stimulation, the upregulated processes also included multiple immune system-related processes *e*.*g*. *antigen processing and presentation* and *interferon induced signaling* and *exocytosis* (Table 2, Fig 3C and 3E, S3 Table). Interestingly, the GO-annotated term *activation of immune system processes* was downregulated after 48 hours of DTaP stimulation (S3 Table).

These data reveal several differences in the processes induced by plain Al(OH)_3_ or the Al(OH)_3_ containing vaccine DTaP: early activation of the antigen processing and presentation pathways after Al(OH)_3_ stimulation, with a delayed response upon DTaP stimulation. Furthermore, DTaP did not induce processes requiring localization, *e*.*g*. exocytosis after 24 hours of stimulation and less strong, compared to Al(OH)_3_ after 48 hours of stimulation. Finally, re-localization processes were also found to be differentially activated upon stimulation with Al(OH)_3_ (upregulated) or DTaP (downregulated) (S3 Table). Thus, the antigens in a vaccine alter the processes activated, in a cell, by the adjuvant.

### DTaP is a stronger activator of the inflammasome than Al(OH)_3_ alone

Part of the adjuvant effect of Al(OH)_3_ is often assigned to activation of the inflammasome [11, 12, 16]. To determine if the presence of antigens influences this inflammasome activation, transcriptome analysis and an IL-1β ELISA were performed. As was observed previously for stimulation with Al(OH)_3_ alone [16], DTaP stimulation also induced gene expression of the inflammasome-related genes, in particular *IL1R1*. Moreover, DTaP induced the expression of *CASP1* and a trend towards upregulation in *MyD88* expression (Fig 2, S1 Table).

**Fig 4:**
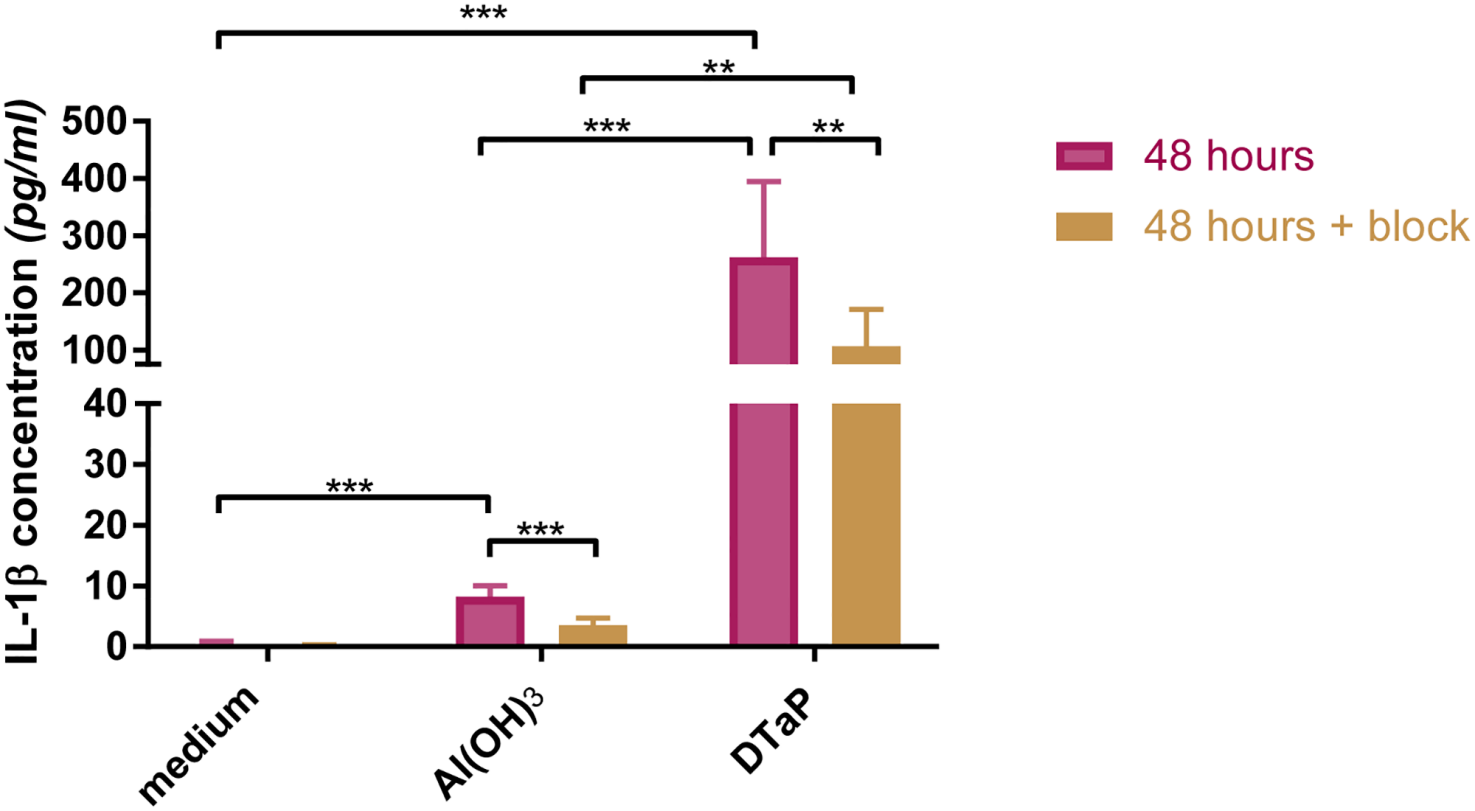
IL-1β secretion profile. Concentrations of IL-1β (average and range) present in supernatants of Al(OH)_3_ or DTaP-stimulated THP-1 cells in the absence or presence of Glybenclamide. Data are from three individual experiments, with two technical replicates. ‘Medium +’ is the PMA-primed medium. Significant values were identified with a *t*-test with a two stage setup method of Benjamini. *p*-Values <0.05 are indicated as * *p*-values <0.01 are indicated as **, whereas *p*-values <0.001 are indicated as ***.

To verify if the enhanced activation after DTaP stimulation resulted in an increased secretion of IL-1β, the presence of IL-1β was measured in culture supernatants of stimulated and PMA-primed THP-1 cells. Medium and medium with PMA do not differ significantly in the induction of IL-1β both with and without blockage, indicating that the prime with PMA does not induce IL-1β secretion [16]. DTaP, however, significantly enhanced the secretion of IL-1β compared to plain Al(OH)_3_ (Fig 4, S4 Table) [16], supporting the transcriptomics data. Subsequently, the role of the inflammasome activation in IL-1β secretion was investigated by adding the inflammasome blocker Glybenclamide during stimulation of the cells. DTaP-induced secretion of IL-1β was dependent on the inflammasome, since 60% of the secretion was inhibited when the inflammasome was blocked (Fig 4), which is less than the inhibition of 80% found in Al(OH)_3_-stimulated cells (Fig 4).

The loss of IL-1β secretion upon inflammasome blockage implies that DTaP, like Al(OH)_3_ depends on the inflammasome for the induction of IL-1β. The substantially higher levels of IL-1β induced by DTaP indicate that the antigens in the vaccine significantly contribute to IL-1β secretion.

### Al(OH)_3_ and DTaP induce different chemokine-related genes

Al(OH)_3_ induces a Th2 polarization [16, 27]. Additionally, the presence of Th1-related and inflammatory cytokines (*IL-2*, *IL-17A* and *IFNγ*) was observed [16] (S1 Table, schematically depicted in Fig 2). DTaP stimulation also induced the Th2 polarization. Increased gene expression of *IL-4* (in one donor with a trend towards upregulation in another donor) and of *IL-5* was found. These expression levels of IL-4 and IL-5 were lower (factor 10 and 2, respectively) than the levels induced by plain Al(OH)_3_ (Fig 2). In addition, DTaP also induced the gene expression of *IL-8* and the anti-inflammatory cytokine *IL-10*. IL-10 represses the formation of IL-2, IL-17A and IFNγ. Transcripts for *IL-2* were a 12-fold less regulated in DTaP-stimulated cells compared to Al(OH)_3_ stimulated cells, while *IL-17A* was even downregulated in DTaP-stimulated monocytes (Fig 2, S1 Table). Expression of *IFNγ* was increased by DTaP stimulation. The gene expression of *CCL2* and *CCL5 w*as induced by DTaP stimulation as was the gene expression of *CCR6* and *CXCR3*.

The data show that Al(OH)_3_ and DTaP differentially regulated the expression of genes involved in the innate immune response. Thus, antigens qualitatively alter the innate immune response induced by Al(OH)_3_ at the level of gene expression towards a less pro-inflammatory profile represented by a decrease in IL-2 and IL-17A gene expression (compared to Al(OH)_3_) and an increase in IL-10 gene expression.

### IL-10 is mainly induced by FHA

**Fig 5:**
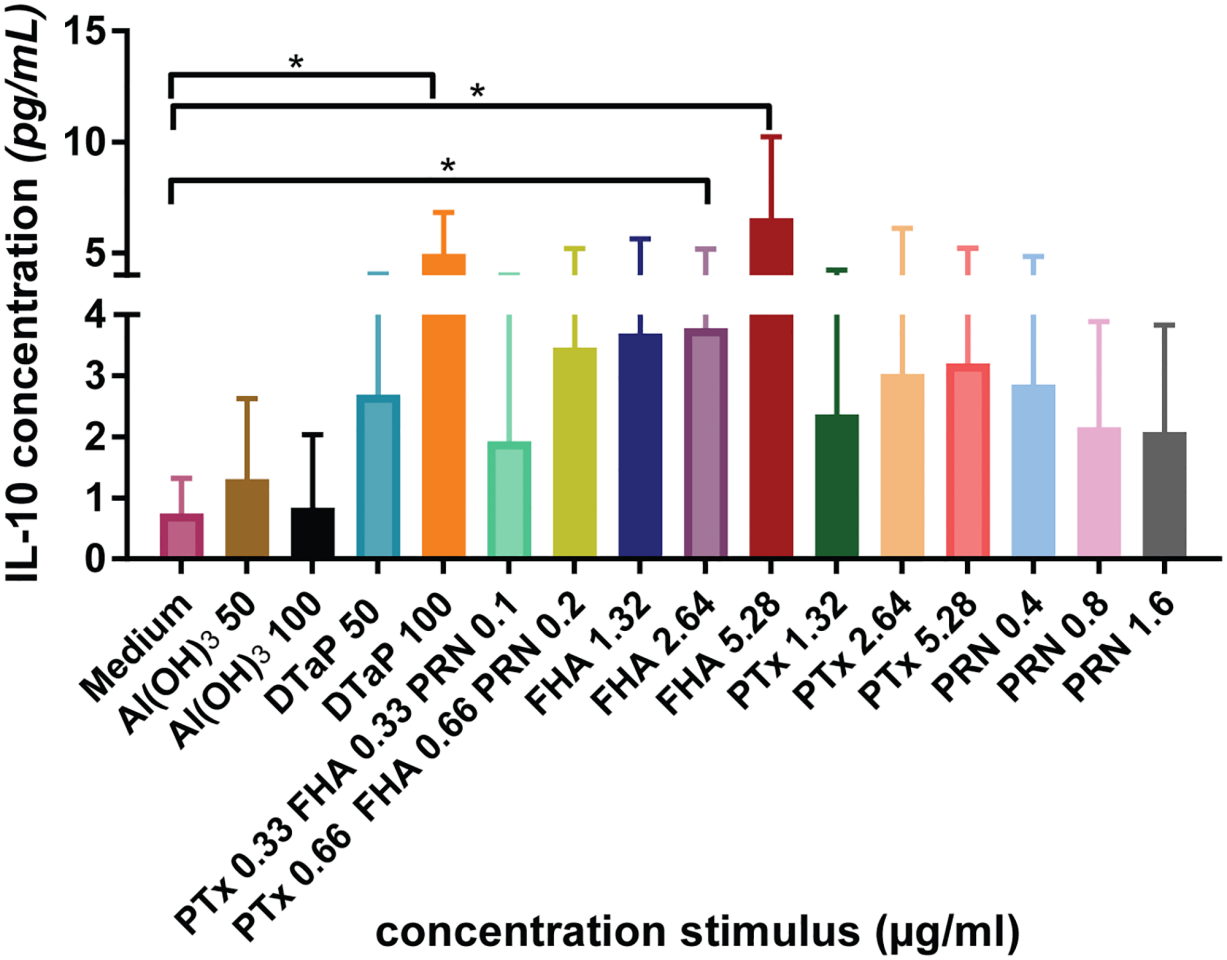
IL-10 secretion profile. The concentration (average and range) of IL-10 induced by the indicated stimulation conditions in pg/ml in THP-1 cells. Data are from three individual experiments. Significant values were identified with a *t*-test with a two stage setup method of Benjamini. *p*-Values <0.05 are indicated as *.

Next, we investigated whether the difference in *IL-10* gene transcription also resulted in increased levels of the IL-10 protein. For this we stimulated PMA-primed THP-1 cells as a monocyte model and IL-10 was measured in the culture supernatant. To identify the antigen responsible for the induction of IL-10, THP-1 cells were stimulated with DTaP, with single antigens of *Bordetella pertussis* that are present in the vaccine, FHA, PTx or PRN, a combination of these antigens without Al(OH)_3_ or with plain Al(OH)_3_ for 48 hours. In accordance with the transcriptome data, DTaP induced the secretion of IL-10, whereas plain Al(OH)_3_ did not. In addition, the combination of antigens without Al(OH)_3_ also enhanced the secretion of the anti-inflammatory cytokine IL-10. FHA was the only individual antigen that induced significant amounts of IL-10 (Fig 5, S5 Table); PRN and PTx contributed marginally.

### DTaP induces type I interferons and IFNγ, but downstream signaling is stronger in monocytes stimulated with plain Al(OH)_3_

**Fig 6:**
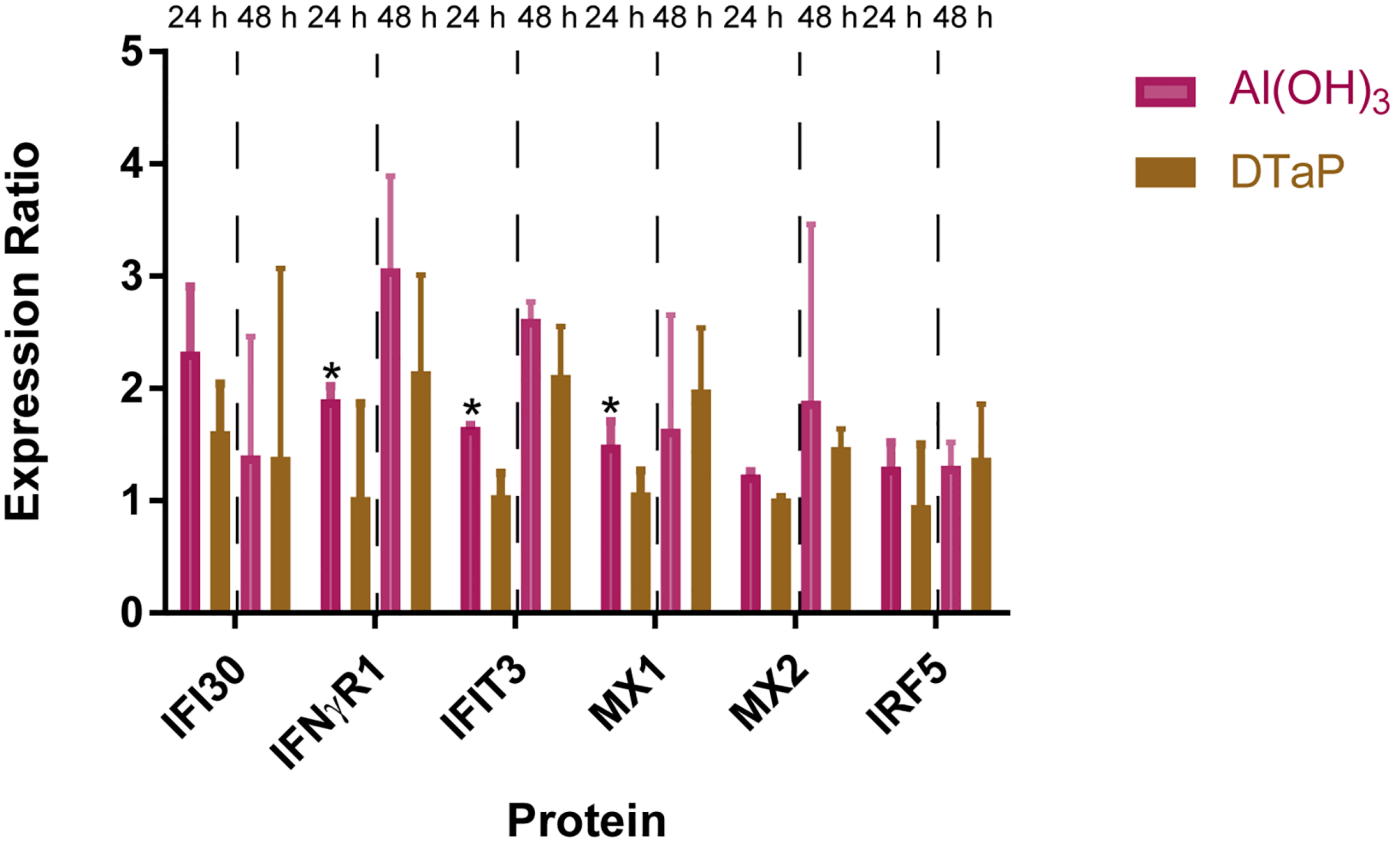
Interferon-related protein expression ratios of stimulated monocytes. The protein expression ratios (median and range of three biological replicates) of indicated interferon-related proteins normalized to medium control (1.0) after 24 and 48 hours of Al(OH)_3_ or DTaP stimulation are depicted. Significance of difference is determined with a *t*-test with a two stage setup method of Benjamini, *p*-Values <0.05 are denoted as: * when upregulated compared to the other stimulation condition.

After 24 hours of stimulation, both Al(OH)_3_ and DTaP-stimulated monocytes showed a trend towards increased gene expression of the type I interferon *IFNβ*. We showed before that Al(OH)_3_ significantly induced proteins downstream of IFNβ [16]. The expressions of two antiviral proteins MX1 and IFIT3, that are specifically induced by type I interferons [28–33], were significantly lower in DTaP-stimulated monocytes compared to the expression in Al(OH)_3_-stimulated monocytes (*p*-value≤ 0.05) (Fig 6). After 24 hours, DTaP specifically induced gene expression of the receptor for IFNβ, *IFNαR1*.

After 48 hours, gene expression levels of all IFN-related genes were reduced to levels lower than naïve cells in both DTaP and Al(OH)_3_-stimulated cells (Table 3). However, proteins downstream of IFNβ were still upregulated: MX1 and IFIT3 in both stimulation conditions and MX2 specifically in Al(OH)_3_-stimulated monocytes (a factor 1.44 stronger compared to DTaP). DTaP specifically induced IRF5, a transcription factor of type I interferons, a 1.43-fold stronger compared to Al(OH)_3_ (Fig 6, Table 1). Although both proteins were not significantly regulated compared to the other stimulation condition a clear trend was observed.

**Table 3:** Fold changes of interferon-related genes relative to control after 48 hours of stimulation.

Gene	DTaP fold change 48 hours	Al(OH)_3_ fold change 48 hours
IFNα	0.47	0.60
IFNαR1	0.76	0.62
IFNβ	0.13	0.11
IFNγ	1.34 (2 times up one time down)	1.42
IFNγR1	0.16	0.15

Data are depicted as an average from three biological replicates.

With respect to type 2 interferons, *IFNγ* gene expression was induced by Al(OH)_3_, as was the expression of the downstream proteins IFI30 and IFNγR1 [16]. DTaP stimulation also induced the gene expression of IFNγ, however, unlike Al(OH)_3_, DTaP did not induce the expression of IFNγ-induced proteins (Table 1). The protein expression of *IFNγR1* was more than a 1.5-fold lower and the expression IFI30 was a 1.48-fold lower in DTaP-stimulated monocytes compared to the effect of Al(OH)_3_ (Table 1, extracted from S2 Table). In addition, after 24 hours, DTaP specifically induced the gene expression of *IFNγR1* in monocytes (Fig 2).

These data provide evidence that Al(OH)_3_ alone as well as formulated in DTaP induce *IFNβ* and *IFNγ* gene expression. However, downstream signaling is impacted by the antigens in DTaP, since this downstream signaling was not evident for IFNβ after 24 hours of DTaP stimulation, while this was the case in Al(OH)_3_-stimulated monocytes. In addition, signaling downstream of IFNγ was induced much stronger in monocytes stimulated by plain Al(OH)_3_.

### Antigens in DTaP do not enhance antigen processing and presentation pathways compared to induction by Al(OH)_3_

Antigen processing and presentation by HLA class II and HLA class I is crucial for the activation of CD4 helper T-cells and cytotoxic CD8 T-cells, respectively. The process of antigen processing and presentation was enriched after 24 hours of Al(OH)_3_ stimulation [16]. Notably, this was not the case after 24 hours of DTaP stimulation, determined upon pathway analysis of the upregulated proteins (Fig 3). DTaP induced the protein expression of HLA-E after 24 hours, but not the expression of HSP90, while Al(OH)_3_ did. The expression of HLA-A and HM13 was enhanced after 48 hours of DTaP stimulation similar to the response induced by Al(OH)_3_ alone (Table 1, S2 Table). Additionally, as for Al(OH)_3_ the gene expression of *HLA-A* was increased by DTaP (S2 Table, summarized in Table 1) [16]. Moreover, DTaP induced the gene expression of *HLA-E* after 24 hours of stimulation (Fig 2, S2 Table).

Various proteins related to antigen processing and presentation were increased upon Al(OH)_3_ stimulation, *i*.*e*. Cathepsin D, Cathepsin L and Legumain [16]. DTaP also induced the protein expression of Cathepsin D and Cathepsin L, with the latter showing a modest increase in expression as compared to Al(OH)_3_ (S2 Table summarized in Table 1). Contrary to the Al(OH)_3_ stimulus, the expression of Legumain, a protease involved in antigen presentation by HLA class II [34–36], was not increased upon DTaP stimulation. After 48 hours, both stimulation conditions increased the protein expression of Cathepsin S (involved in HLA class II antigen presentation) and Legumain, while Cathepsin B was only induced by DTaP, also compared to Al(OH)_3_. The similar strength in the induction of antigen processing and presentation pathways and associated proteins, indicates that Al(OH)_3_ adjuvant alone is sufficient to activate antigen processing and presentation pathways and that the antigens in DTaP do not enhance these processes further.

## Discussion

Aluminum salts have been used as adjuvants in a wide variety of vaccines and these formulations are known to induce a Th2-biased response. In this study, we investigated the immune skewing of one such vaccine, DTaP, in detail and compared the *in vitro* innate immune response with those initiated by a plain Al(OH)_3_ stimulus as described in our previous study [16]. Combined proteomics and transcriptomics analysis revealed several similarities between the monocyte responses towards the vaccine and the plain Al(OH)_3_ adjuvant, like activation of the inflammasome. Flow cytometry analysis could not be used in the presence of an Al(OH)_3_ suspension because the particles cause a substantial background masking the specific signals. Nevertheless, predominant differences were found with respect to interferon and IL-10 signaling: (*i*) the induction of the anti-inflammatory cytokine IL-10 by DTaP, (*ii*) gene expression of the pro-inflammatory cytokines *IFNγ*, *IL-2* and IL-17A by Al(OH)_3_, (*iii*) differences in the production of IFN-induced proteins between stimulation conditions and (*iv*) processes involved in re-localization of proteins and macromolecules being induced by Al(OH)_3_, but downregulated by DTaP, which could be related to the induction of exocytosis and endocytosis, respectively. However, the implications of this difference for the functional immune response need further investigation.

**Fig 7:**
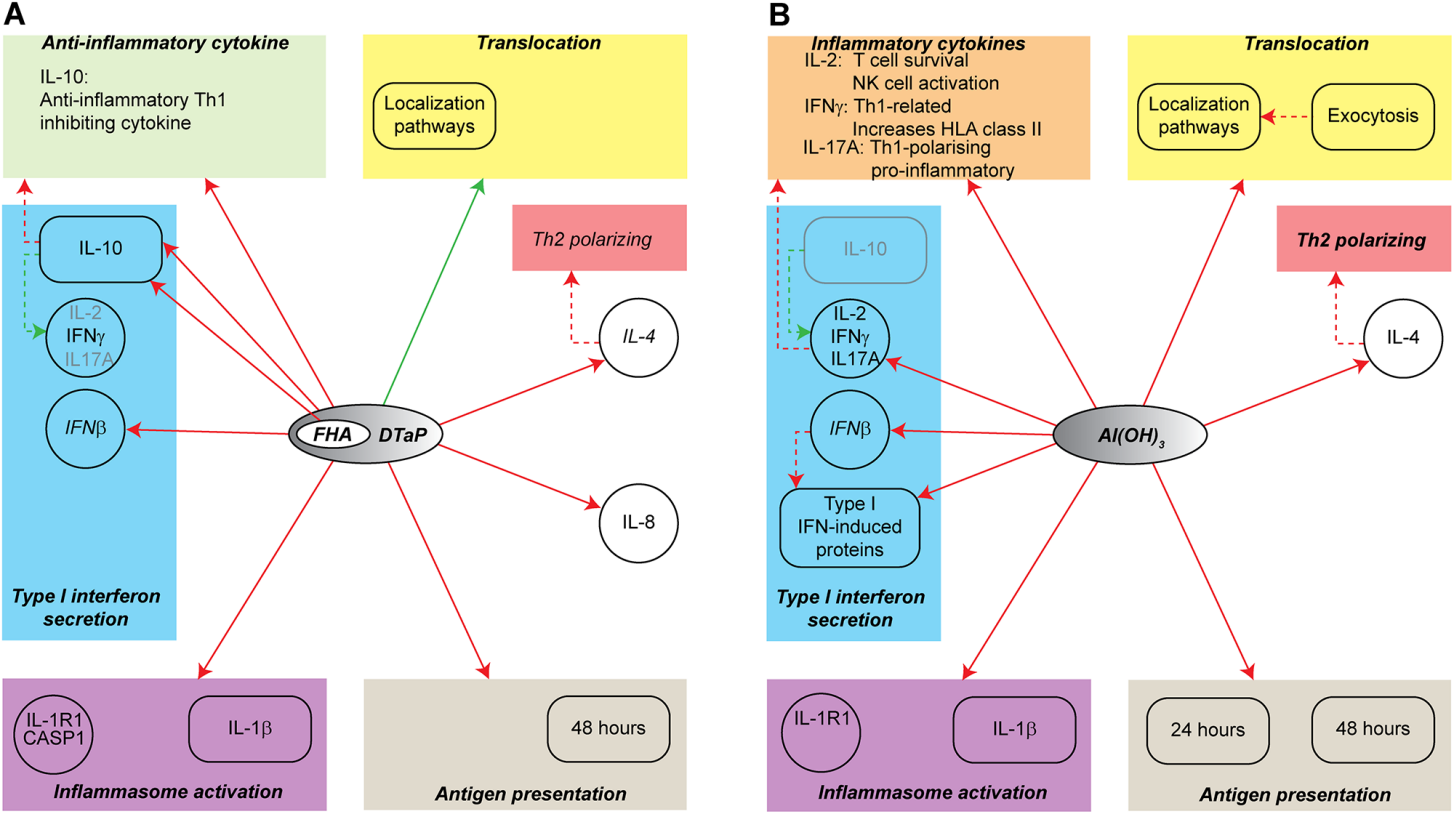
Overview of all data. The different effects of Al(OH)_3_ and DTaP stimulation on monocyte functions are summarized. The red arrows from a stimulation DTaP and FHA (A) or Al(OH)_3_ (B) represent an upregulation, the green arrows represent a down regulation. Red arrows towards a box indicate that the genes/proteins in the box are upregulated. The title in the box represents the process regulated. For interferon secretion, individual arrows indicate if genes or processes are upregulated. The dashed arrows represent connections based on the literature. The circles represent measurements at the gene expression level while the rounded boxes represent measurements at the protein level. The green and orange rectangles represent consequences of an inhibition or activation by one of the stimulations.

The unique induction of *IL-2* and *IL-17A* by plain Al(OH)_3_ and the unique induction of IL-10 by DTaP (annotated as differences (*i*) and (*ii*) in the previous paragraph, respectively) are likely related, since IL-10 inhibits the formation of IL-2, IL-17A and IFNγ [37–43] (Fig 7). Note: IFNγ was equally induced by both stimulation conditions. The induction of IL-10 by DTaP could be attributed to the *B*. *pertussis* antigens present in the vaccine and more specifically to FHA with a minor contribution for PRN; this is in agreement with previously described data [18, 44–46].

IL-2 and IL-17A are important pro-inflammatory cytokines in the protective response towards bacteria and viruses. IL-2 is involved in activating and steering NK cells and is required for T-cell survival [47–49]. IL-17A is a pro-inflammatory cytokine involved in cell trafficking and inflammation and induces in innate immune cells IL-12 secretion, resulting in Th1 polarization [50, 51]. IFNγ plays a role in the induction of HLA class II expression on the cell surface and is related to the polarization of a Th1 response partly by the inhibition of a Th2 response [52–55]. By inducing IL-10, the monocyte response to DTaP might have become less inflammatory and less Th1-related. For bacteria, the secretion of anti-inflammatory components, such as the *B*. *pertussis* antigen FHA, can be very effective in evading the human immune response. To improve current DTaP vaccines it may be relevant to select the antigenic composition not only based on immunogenicity but also on innate immune modulating effects of the antigens.

The third difference, the specific upregulation of IFNβ-induced proteins upon Al(OH)_3_ stimulation could be related to the induction of IL-10 by DTaP, since IFNβ can be consumed by monocytes to produce IL-10 [56]. This results in an apparently limited upregulation of IFNβ-induced proteins as observed after DTaP stimulation, as well as the increased IL-10 secretion. The proteins downstream of IFNβ are all proteins involved in the defense response against viruses, thus perhaps not functional in the response against the bacterial antigens in the DTaP vaccine. This explains why these are not formed upon DTaP stimulation and provide evidence that the specific response against the antigens of DTaP can antagonize the underlying broad spectrum innate response against the adjuvant.

As described previously, Al(OH)_3_ stimulation of monocytes induced re-localization processes [16]. In DTaP-stimulated monocytes, these processes were downregulated after 24 hours of stimulation. Processes requiring protein localization include *secretion*, *exocytosis*, *vesicle mediated transport*, *communication* and *signal transduction* [57]. Al(OH)_3_ did induce processes requiring localization after 24 and 48 hours of stimulation, whereas in DTaP-stimulated monocytes the processes requiring localization were not evident after 24 hours of stimulation. Upon prolonged stimulation, these particular processes were enriched in DTaP stimulated monocytes. However, the enrichment was less strong compared to Al(OH)_3_-stimulated monocytes, implicating that this could partly explain the differences in localization processes. In contrast to the secretory pathways, DTaP specifically induced the process of endocytosis after 24 hours. Endocytosis plays a role in antigen processing and presentation and cross-presentation [58, 59], which is unlikely occur in the cells where no exogenous antigen is present; this is confirmed by the stimulation with plain Al(OH)_3_ after which we did not observe enhanced endocytosis [16].

Processes induced by both Al(OH)_3_ and DTaP are activation of the inflammasome and the secretion of IL-1β [16], however, much stronger by DTaP than by Al(OH)_3_. This implies that the antigens in the vaccine boost the secretion of IL-1β (Fig 7). IL-1β is a cytokine involved in many innate immune system-related processes, *e*.*g*. the induction of adhesion molecules on the cell surface, being co-stimulatory for T cells and induce Th17 polarization [60, 61]. The stronger induction of IL-1β by DTaP most likely results in a stronger co-stimulation for T cells and induction of the adaptive immune response.

Other processes being regulated both by Al(OH)_3_ and DTaP are the activation of complement and antigen processing and presentation. Al(OH)_3_ stimulation alone is enough to induce proteins related to antigen processing and presentation by both HLA class I and class II [16]. This induction is not affected further when the complete vaccine is used as a stimulus (Fig 6). This indicates that Al(OH)_3_ plays a role in antigen processing and presentation previously thought to be related to antigens.

These *in vitro* data show that we indeed find the described Th2 profile often assigned to Al(OH)_3_ and Al(OH)_3_-adjuvanted vaccines, like DTaP [8, 62, 63]. However, our findings clearly show that the effect of the adjuvant can be influenced significantly by the antigens in the vaccine, since Al(OH)_3_ induced a mixed Th1/Th2 profile and that the antigens in the vaccine formulation influence this to a great extent. This difference, the mixed response we observe for Al(OH)_3_ compared to the Th2 response described in literature [27], could be related to responses in mice *versus* human: in mice, Al(OH)_3_ is specifically a Th2 polarizing adjuvant while in human it induces a more mixed innate response [8, 46–49]. These differences between *in vivo* mice data and *in vitro* human data indicate that our model of human monocytes can be important in the translation of *in vivo* mice to human data. However, in this analysis only one cell type was used to determine the induced immune responses, thus we would miss interactions between different cell types, involved in the immune response. An additional consideration could be that for some proteins their functions are not annotated, thus the link to the pathways they would have been involved in. The comprehensive systems approach allows for the identification of multiple differences in innate pathway activation in monocytes between an adjuvant alone and a complete adjuvanted vaccine. Our results show that antigens can have a profound impact on the adjuvant activity of a vaccine. A possible explanation could be that DTaP contains Al(OH)_3_ particles with different physico-chemical interactions due to inhomogeneous antigen distribution. For example, bare Al(OH)_3_ particles may exist next to particles with adsorbed antigen, or the different antigens are adsorbed on different particles in case the formulation of the final bulk is done by mixing pre-adsorbed individual antigens. This is unlikely since studies have shown that any inhomogeneous distribution is equilibrated due to rearrangements of the Al(OH)_3_ primary particles and/or antigen [64]. Most likely, the distinct innate effects between plain Al(OH)_3_ and an Al(OH)_3_-adjuvanted vaccine are caused by intrinsic adjuvant actions of some antigens present, *e*.*g*. the ability of FHA to induce IL-10 as shown here and by others [65]. Our study clearly reveals that the combination of antigen and the adjuvant determine the innate effects caused by a vaccine.

## Supporting information

S1 Fig Flow cytometry data gating staining. Data of one representative donor to illustrate the gating strategy used: (A) represents ungated monocytes, in (B) the live cells are gated, (C) represents the single stained cells inside the live cells, (D) represents the monocytes inside the single stained cells and (E) is a histogram of CD80-stained cell in which the grey line represents medium control and the red line represents LPS as a positive control.
(TIF)

S1 Table qPCR raw data table. Values are Ct values, Delta Ct values, DeltaDelta Ct values and ratios to control for a given donor and stimulation condition (Al(OH)_3_ or DTaP) after 24 hours of stimulation. The technical replicates used for the determination of the significance threshold are also depicted in the tab ‘Technical replicates’. A heap map of all the individual donors is depicted in the tab ‘heatmap individual donors’.
(XLSX)

S2 Table LC-MS/MS raw data table. LOG2 ratios as obtained by Proteome Discoverer for the individual proteins are depicted. To combine multiple entries for the same protein, columns B and C contain the information used to remove redundancy in protein accession numbers (column A). Blank values indicate that no quantification data were available. The tab ‘Regulated Proteins’ contains the proteins that were regulated with at least a factor 1.5 in one of the stimulation conditions, after pooling and normalization. The fold changes are depicted as LOG(2) factors.
(XLSX)

S3 Table Enriched pathways and GO terms. The pathways that are enriched after a specific stimulation condition and time point. The pathways are GO terms or KEGG pathways, which are enriched after one of the stimulation conditions with an FDR of <0.1. The proteins were at least a factor 1.5 regulated compared to control in 2 out of 3 donors.
(XLSX)

S4 Table Raw IL-1β ELISA. Measured IL-1β concentrations in pg/μl for the individual experiments.
(XLSX)

S5 Table Raw IL-10 ELISA. Measured IL-10 concentrations in pg/μl for the individual experiments.
(XLSX)
