# Supplementary figures and images for: Vaccine antigens modulate the innate response of monocytes to Al(OH)3
Source: PLoS One. 2018 May 29;13(5):e0197885. doi: 10.1371/journal.pone.0197885 (PMC5973561; doi:10.1371/journal.pone.0197885)

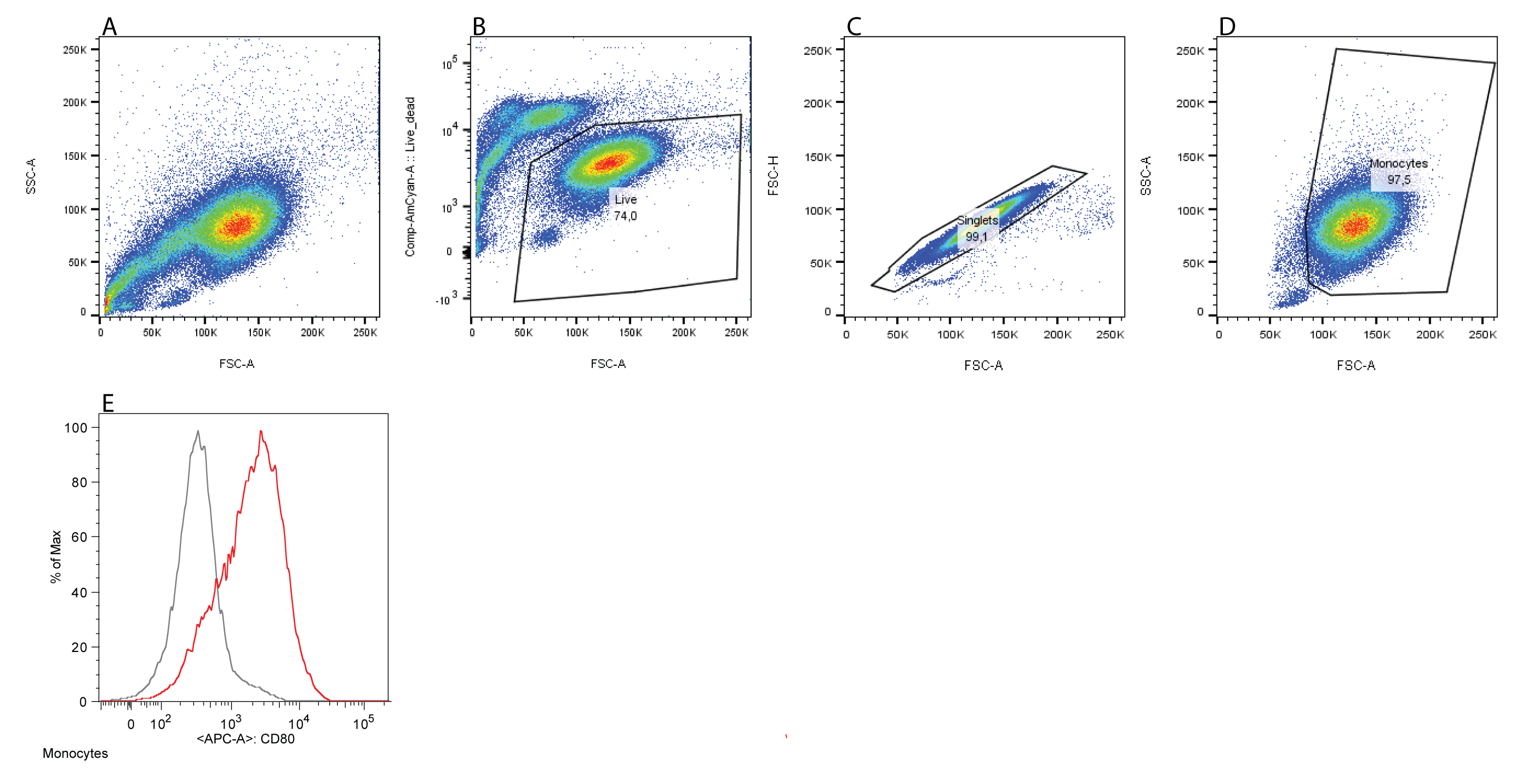

Supplement: S1 Fig — Data of one representative donor to illustrate the gating strategy used: (A) represents ungated monocytes, in (B) the live cells are gated, (C) represents the single stained cells inside the live cells, (D) represents the monocytes inside the single stained cells and (E) is a histogram of CD80-stained cell in which the grey line represents medium control and the red line represents LPS as a positive control. (TIF) [file pone.0197885.s001.tif]
